# Radioprotective Effects of Carvacrol and/or Thymol against Gamma Irradiation-Induced Acute Nephropathy: In Silico and In Vivo Evidence of the Involvement of Insulin-like Growth Factor-1 (IGF-1) and Calcitonin Gene-Related Peptide

**DOI:** 10.3390/biomedicines11092521

**Published:** 2023-09-13

**Authors:** Yasmen F. Mahran, Layla A. Al-Kharashi, Reem T. Atawia, Rawan Turki Alanazi, Amal M. Bin Dhahi, Rawd Alsubaie, Amira M. Badr

**Affiliations:** 1 Department of Pharmacology and Toxicology, Faculty of Pharmacy, Ain Shams University, Cairo 11566, Egypt; reemtarekatawia@pharma.asu.edu.eg (R.T.A.); amibadr@ksu.edu.sa (A.M.B.); 2 Department of Pharmacology and Toxicology, College of Pharmacy, King Saud University, Riyadh 11211, Saudi Arabia; lalkharashi@ksu.edu.sa; 3 Department of Pharmaceutical Sciences, College of Pharmacy, Southwestern Oklahoma State University, Weatherford, OK 73096, USA; 4 Student, Pharmacy College, King Saud University, Riyadh 11211, Saudi Arabia; rawan1939@gmail.com (R.T.A.); aldhahiamal@gmail.com (A.M.B.D.); rawd.alsubaie@gmail.com (R.A.)

**Keywords:** γ-irradiation, radiotherapy nephropathy, carvacrol, thymol, acute nephrotoxicity, CGRP, IGF-1

## Abstract

Radiotherapy (RT) is an effective curative cancer treatment. However, RT can seriously damage kidney tissues resulting in radiotherapy nephropathy (RN) where oxidative stress, inflammation, and apoptosis are among the common pathomechanisms. Carvacrol and thymol are known for their antioxidative, anti-inflammatory, and radioprotective activities. Therefore, this study investigated the nephroprotective potentials of carvacrol and/or thymol against gamma (γ) irradiation-induced nephrotoxicity in rats along with the nephroprotection mechanisms, particularly the involvement of insulin-like growth factor-1 (IGF-1) and calcitonin gene-related peptide (CGRP). Methods: Male rats were injected with carvacrol and/or thymol (80 and 50 mg/kg BW in the vehicle, respectively) for five days and exposed to a single dose of irradiation (6 Gy). Then, nephrotoxicity indices, oxidative stress, inflammatory, apoptotic biomarkers, and the histopathological examination were assessed. Also, IGF-1 and CGRP renal expressions were measured. Results: Carvacrol and/or thymol protected kidneys against γ-irradiation-induced acute RN which might be attributed to their antioxidative, anti-inflammatory, and antiapoptotic activities. Moreover, both reserved the γ -irradiation-induced downregulation of CGRP- TNF-α loop in acute RN that might be involved in the pathomechanisms of acute RN. Additionally, in Silico molecular docking simulation of carvacrol and thymol demonstrated promising fitting and binding with CGRP, IGF-1, TNF-α and NF-κB through the formation of hydrogen, hydrophobic and alkyl bonds with binding sites of target proteins which supports the reno-protective properties of carvacrol and thymol. Collectively, our findings open a new avenue for using carvacrol and/or thymol to improve the therapeutic index of γ-irradiation.

## 1. Introduction

Worldwide, cancer incidence and mortality are increasing rapidly. According to GLOBOCAN 2020, 19.3 million new cancer cases and almost 10 million cancer deaths were reported in 2020 [1,2]. One of the most critical forms of curative cancer treatment is radiotherapy (RT), used alone or in conjunction with chemotherapy and/or surgery [3,4,5] in more than half of all cases [6,7]. Ionizing radiation is widely recognized to damage DNA molecules in the cell, including the formation of single- or double-strand breaks (DSBs) and cross-link, leading to the eradication of cancer cells [8]. Despite its effectiveness in controlling tumor growth and prolonging patient survival rates, RT can seriously damage healthy tissues that are unavoidably exposed to radiation, resulting in lower doses being applied and suboptimal tumor control. Although significant progress has been made in this field over the past century, one of the major challenges in radiation therapy is selectively targeting cancerous tissues [9,10,11].

Kidneys are the most radiosensitive abdominal organ with a documented tolerance dose of 20 Gy [12]. Increased blood permeability, perfusion problems, inflammation, and fibrosis are among the common features of radiotherapy nephropathy (RN) [13,14]. In most cases, acute nephrotoxicity evolves into chronic kidney disease, with symptoms including fatigue, malignant hypertension, edema, azotemia, and shortness of breath within six to twelve months of exposure [15]. To date, the molecular pathomechanisms responsible for RN are not fully understood; however, biomarkers for diagnosing and assessing RN progression and severity still remain a challenge [10]. Among the documented pathomechanisms of RN are DNA damage-mediated cell death [16], cellular senescence [17], oxidative stress, and inflammation [18,19]. Despite dose-fractionation attempts, radiation-induced kidney insults cannot be completely eradicated. In these cases, prophylactic administration of radioprotectants before irradiation may be a viable alternative [20].

Moreover, calcitonin gene-related peptide (CGRP) is a potent neuropeptide that promotes systemic vasodilation and plays a crucial role in migraine, pain transmission, and cardiovascular homeostasis [21]. Also, CGRP, through its peripheral receptors, affects renal hemodynamics by increasing renal cyclic AMP levels in a dose-dependent manner [22,23]. Recently, Zhong and co-workers suggested that CGRP receptor blockade with CGRP8-37 worsened ischemia/reperfusion-mediated kidney inflammation and injury [24]. Moreover, the depletion of insulin-like growth factor-1 (IGF-1) has been shown to play a role in radiation- and chemotherapy-induced kidney insults [25,26]. IGF-1 upregulation has been linked mutually to the CGRP signaling in some settings [27,28]; however, the involvement of this crosstalk in the nephroprotective mechanisms has not been elaborated.

Thymol, and its phenol isomer, carvacrol, are the main natural terpenoids of *Thymus vulgaris* essential oil. Both components are known for their antioxidative, anti-inflammatory, antiapoptotic, and antimicrobial activities [29,30,31]. In addition, radioprotective effects of thymol and carvacrol have been documented in different ionizing radiation-induced injuries [32,33,34,35,36]. Studies showed that carvacrol and thymol have some promising effects against chemotherapy-induced nephrotoxicity through antioxidative, antiapoptotic, anti-inflammatory, and cytoprotective mechanisms [37,38,39,40]. Carvacrol has been reported to be a ligand for the transient receptor potential channel ankyrin 1 (TRPA1), which mediates the expression and release of CGRP [41,42]. Therefore, the aim of this study is to investigate the potential nephroprotective effects of carvacrol and/or thymol against gamma irradiation-induced nephrotoxicity in rats and to explore the involvement of CGRP signaling and IGF-1 in the nephroprotection mechanisms.

## 2. Material and Methods

### 2.1. Drugs and Reagents

Carvacrol (2-hydroxy-4-cymene; isothymol) was obtained from Extrasynthese Co. (Z.I. Lyon Nord, Genay Cedex, France) and thymol (2-isopropyl-5-methylphenol) was obtained from Abcam, Cambridge, UK. Other reagents and solvents were of the highest grade commercially available.

### 2.2. Radiation Facility

Using a Gamma Cell-40 biological irradiator with a Cesium (^137^CS) source located at the Research Centre of King Saud University, Riyadh, Saudi Arabia, rats were exposed to a single dose of whole-body γ-irradiation (6 Gy) at a dose rate of 0.48 Gy/min to induce kidney damage. This dose was chosen according to previous studies [14,20]. Rats were placed in plastic boxes in a chamber attached to the irradiation equipment.

### 2.3. Animals and Treatments

Mature male Wistar rats (150–200 g body weight) at 8 weeks old were obtained from the animal house of the Faculty of Pharmacy, King Saud University. To provide standard laboratory conditions, rats were acclimatized upon arrival and housed for one week (4 per cage) in a controlled animal holding room with a 12/12 h light/dark cycle. Also, temperature (25 °C) and relative humidity were always monitored. In accordance with standard guidelines, standard pellet diets containing at least 20% protein, 5% fiber, 3.5% fat, 6.5% ash, and a vitamin mixture were used. The study was conducted according to the ethical guidelines of the Faculty of Pharmacy, King Saud University, Saudi Arabia (IRB number; KSU-SE-23-03).

Animals were randomly divided into five groups, eight rats each. Group 1 (control group): normal non-irradiated rats were injected intraperitoneally (i.p.) with 1 mL vehicle (0.5% DMSO in normal saline) for five days. Group 2 (irradiated group, R): rats were injected i.p. with 1 mL vehicle (0.5% DMSO/normal saline) for five days and exposed to radiation at a dose level of 6 Gy on day three and sacrificed after two days. Group 3 (irradiated + carvacrol group, R+V): rats were injected i.p. with carvacrol (80 mg/kg BW in the vehicle) for five days starting three days before irradiation (single dose of 6 Gy) and sacrificed two days after irradiation. Group 4 (irradiated + thymol group, R+T): rats were injected i.p. with thymol (50 mg/kg BW in the vehicle) for five days starting three days before irradiation (single dose of 6 Gy) and sacrificed two days after irradiation. Group 5 (irradiated + carvacrol+ thymol group, R+T+V): rats were injected i.p. with both carvacrol and thymol (80 and 50 mg/kg BW in the vehicle, respectively) for five days starting three days before irradiation (single dose of 6 Gy) and sacrificed two days after irradiation. In accordance with previous studies, we chose carvacrol and thymol doses [33,43,44]. These doses do not show any signs of toxicity according to the documented data of lethal dose 50 (LD50) of thymol and carvacrol in rats [45,46]. In addition, carvacrol and thymol are listed by the United States Food and Drug Administration as ‘Generally Recognized As Safe’ for use as a food additive; therefore, they are considered to be safe with negligible toxicity [47,48].

Rats were weighed daily until the day of sacrifice. On this day, animals were fasted for 12 h, exposed to progressively higher concentrations of CO_2_, and decapitated. Following scarification, kidney samples were collected and divided into two portions. The first portion was homogenized and centrifuged at 10,000× *g* for 15 min, then stored at −80 °C for the assessment of oxidative stress biomarkers, pro-inflammatory mediators, IGF-1, and CGRP. Blood was collected from the trunk and centrifuged for 30 min at 4 °C and 2000 rpm. Histopathological examination was performed on the second portion of kidney samples fixed in 10% neutral formalin.

### 2.4. Assessment of Serum Urea and Renal Oxidative Stress Biomarkers

We assessed blood urea nitrogen colorimetrically using the commercially available kits of United Diagnostic Industry (UDI, Damam, Saudi Arabia) according to the manufacturer’s instructions. We measured the oxidative stress markers for kidney tissue homogenates including reduced glutathione (GSH) and malondialdehyde (MDA). First, according to Beutler et al. [49], measurement of GSH involves reducing 5,50-dithiobis-2-nitrobenzoic acid with GSH and measuring its absorbance at 412 nm. Secondly, as described by Ohkawa et al. [50], MDA concentration can be used as an indicator of lipid peroxidation. The assay was based on measurements of thiobarbituric acid reactive (TBAS) species, and an absorbance of 532 nm was measured for the resulting pink material.

### 2.5. Histopathological Examination

We fixed kidney tissues of the different groups in 10% neutral-buffered formalin. Then, the specimens were dehydrated and embedded in molten paraffin. Using a rotary microtome, 4-micron-thick sections were cut and mounted on clean slides. Then, sections were stained with hematoxylin and eosin (H&E) for light microscopic histological examination [51] and photos were taken using a digital video camera mounted on a light microscope (DX 72, Olympus, Tokyo, Japan).

### 2.6. Assessment of Renal Inflammatory and Apoptotic Biomarkers

Using the commercially available kits and following the manufacturer’s instructions, we evaluated the effects of ionizing radiation and carvacrol and/or thymol on the expression of some pro-inflammatory mediators in the kidney. Nuclear factor kappa B (NF-κB) was assayed using the NF-κB ELISA Kit (Sigma Aldrich, St. Louis, MO, USA) and tumor necrosis factor- alpha (TNF-α) was measured using the TNF-α ELISA Kit (Sigma Aldrich, USA).

Immunohistochemistry (IHC) examination of Caspase-3 was carried out according to a standard protocol in which the kidney paraffin blocks were deparaffinized, rehydrated and then incubated with the primary antibody against, rat Caspase-3 (1:100), purchased from Thermo Fisher Inc., Waltham, MA, USA, then followed using the suitable secondary antibody Envision kit (DAKO), and washed with phosphate buffer saline (PBS). Finally, sections were incubated with diaminobenzidine (DAB) for 10 min, washed again with PBS, and counterstained with Mayer’s hematoxylin for examination under a light microscope. The quantitative expression of Caspase-3 in kidney tissues was calculated as area % of immunoexpression levels of Caspase-3 in six random non-overlapping fields/sections using the Leica Application module attached to a Full HD microscopic imaging system (Leica Microsystems GmbH, Wetzlar, Germany).

### 2.7. Assessment of IGF-1 and CGRP

Using the commercially available kits Rat CGRP ELISA Kit (AFG Bioscience, Northbrook, IL, USA) and Rat IGF-1 ELISA Kit (Cusaibo, Wuhan, China), we evaluated the effects of ionizing radiation and carvacrol and/or thymol on the renal expression of IGF-1 and CGRP.

### 2.8. Molecular Docking

PDB files of the TNF-α, NF-κB1, p65, CGRP and IGF-1 were downloaded from the RSCB PDB online platform (https://www.rcsb.org/) and Uniprot. Proteins were selected with one or more cocrystallized ligands and crystal structures with small “resolution” value. The 3D structures of carvacrol and thymol were obtained from the PubChem database (https://pubchem.Ncbi.Nlm.Nih.Gov/). Docking validation after hydrogenation and dehydration was performed by AutoDockTools (version 1.5.6) and AutoDock Vina (version 1.1.2) and docking pocket regions were predicted by DeepSite software (https://www.Playmolecule.Com). Results were visualized into BioVia2020 software.

### 2.9. Statistical Analysis

The statistical analysis was computed using the Graph-Pad Prism 5 (Graph-Pad Software, San Diego, CA, USA). Differences between experimental groups were tested by one-way analysis of variance (ANOVA), followed by the Tukey–Kramer multiple comparisons test. *p* < 0.05 was considered significant. Data were presented as mean ± SEM.

## 3. Results

### 3.1. Carvacrol and/or Thymol Ameliorates the γ-Irradiation-Induced Deterioration of Kidney Indices and Oxidative Stress

As shown in Table 1, γ-irradiation induced a significant (approx. 3-fold) increase in serum BUN levels when compared to the control group. Interestingly, carvacrol and/or thymol administration ameliorated the γ-irradiation-induced increase in serum BUN levels compared to that in the irradiated group. In addition, the combination of carvacrol and thymol administration showed no significant difference in serum BUN levels compared to either drug alone (Table 1).

In addition, oxidative stress markers caused significant increment in renal lipid peroxidation levels (150% increase in terms of thiobarbituric acid reactive substances (TBARS)) and severe depletion of the renal GSH content (by about 95%) in the irradiated rats when compared to the control group. The administration of carvacrol and/or thymol significantly reduced the renal TBARS to levels comparable to those of the control group and increased the renal GSH content. The GSH content in the carvacrol- and/or thymol-irradiated group was significantly higher compared to the non-irradiated control: by about 2-fold for carvacrol and thymol alone, and 3-fold for their combination, as shown in Table 1.

### 3.2. Carvacrol and/or Thymol Improves the γ-Irradiation-Induced Deterioration of Renal Histopathology

**Figure 1:**
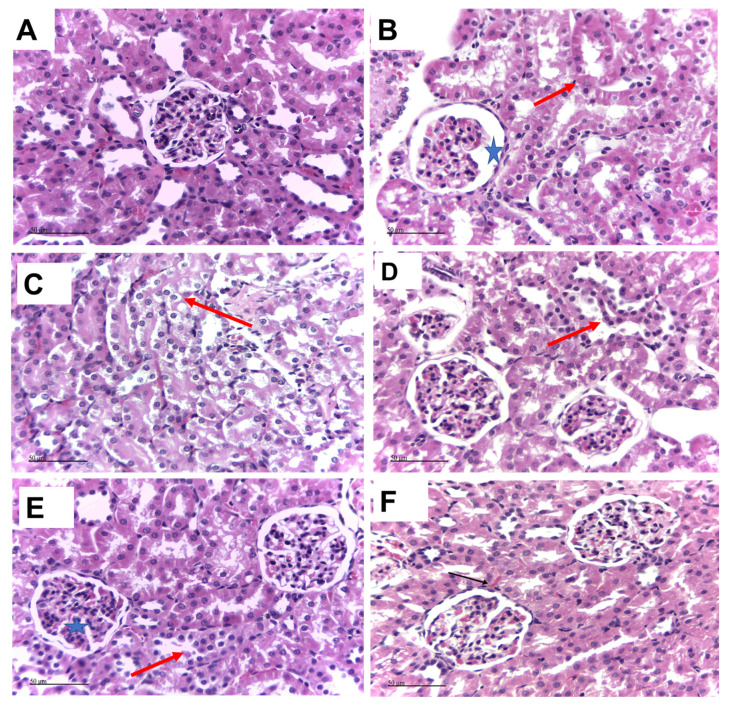
Impact of Carvacrol and/or Thymol Administration on the Histopathological Features of Renal Samples in γ-irradiated Rats. (**A**): Control normal rats; (**B**,**C**): γ-irradiated rats; (**D**,**E**): irradiated rats treated with carvacrol (80 mg/kg) and thymol (50 mg/kg), respectively; (**F**): irradiated rats treated with carvacrol (80 mg/kg) and thymol (50 mg/kg). Control group shows normal histological structure of the renal tissue. Irradiated group shows mild atrophy of renal glomeruli with increased renal spaces, vacuolar degeneration, necrobiotic changes, and karyopyknosis (arrow) (star). (**D**,**E**) groups show milder vacuolar degenerative changes. Carvacrol and thymol irradiation-treated groups show apparently normal renal corpuscles.

As shown in Figure 1, the control group showed normal histological structures of renal tissue with apparently normal intact renal corpuscles and tubular structures in different zones (Figure 1A). On the other hand, the irradiated group demonstrated mild atrophy of renal glomeruli with increased renal spaces (star), with focal areas showing vacuolar degeneration in renal tubular epithelium in different tubular segments (arrow). Scattered tubular epithelial cells showed necrobiotic changes and karyopyknosis (dashed arrow) (Figure 1B,C). Interestingly, in the thymol-treated group, most of the renal corpuscles were more protected with scattered corpuscles showing hypercellularity (star). Milder vacuolar degenerative changes were recorded in most of the tubular epithelium (arrow) when compared with the irradiated kidney sections. However, few luminal epithelial casts (dashed arrow), tubular dilation in some medullary tubular sections (Figure 1 star), and congested intertubular blood capillaries (red arrow) were observed (Figure 1D). Additionally, the carvacrol-treated group showed more protected renal corpuscles, compared to the thymol-treated group. However, more pronounced vacuolar degenerative changes (arrow), as well as luminal casts (dashed arrow), were observed in focal areas in renal tubular epithelium segments. Scattered tubular segments showed karyopyknosis of the lining epithelium (red arrow) (Figure 1E). Furthermore, samples of rats treated with both thymol and carvacrol showed apparently normal renal corpuscles. Tubular segments were protected with variable degrees in different sections. Vacuolar degeneration with necrobiotic changes of the tubular epithelium (arrow) with intraluminal cast formation was detected in some tubular sections (dashed arrow), as shown in Figure 1F.

### 3.3. Carvacrol and/or Thymol Mitigates the γ-Irradiation-Induced Renal Inflammatory and Apoptotic Responses

**Figure 2:**
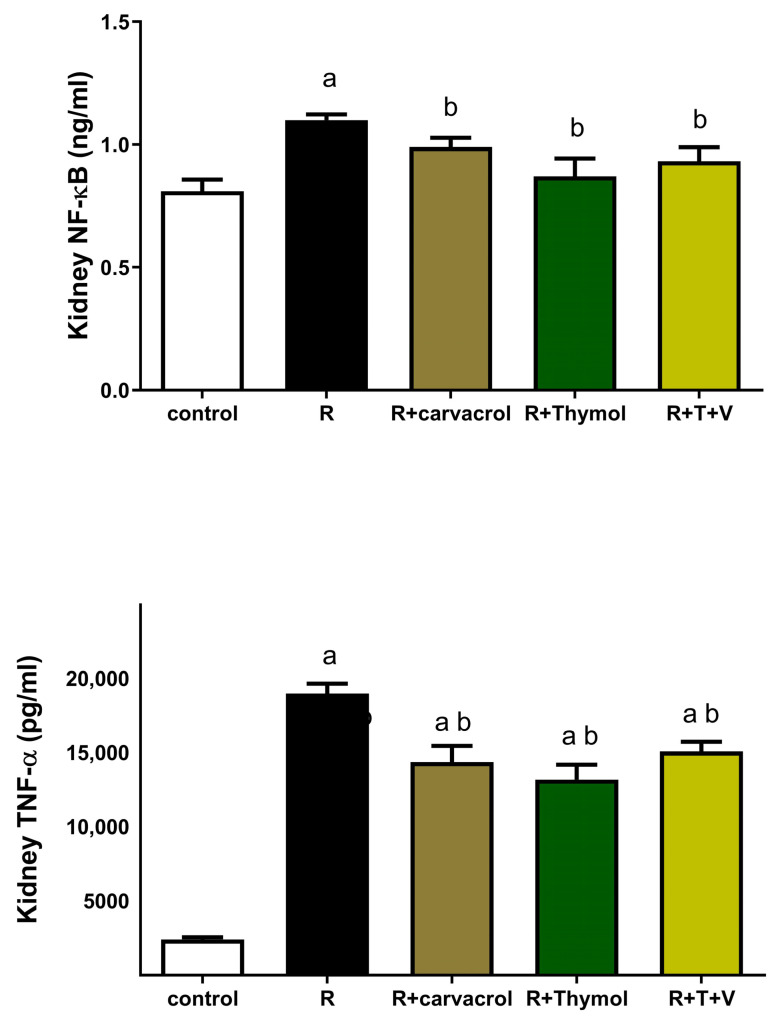
Impact of Carvacrol and/or Thymol on Renal Expressions of Nuclear Factor-kB (NF-kB) and Tumor Necrosis Factor-α (TNF-α) in γ-irradiated Rats. Data are presented as mean ± SEM. ^a^ *p* < 0.05 versus control group, ^b^ *p* < 0.05 versus irradiated group, using one way ANOVA followed by Tukey–Kramer as post hoc test. R: γ-irradiation; V: Carvacrol; T: Thymol.

Two days post irradiation, the renal expressions of both NF-κB and TNF-α were significantly increased to approximately 137% and 800%, respectively, in the irradiated rats when compared with the control rats (Figure 2). On the other hand, the administration of carvacrol and/or thymol three days before irradiation and two days after rradiation significantly ameliorated these changes and decreased the renal expression of NF-κB by about 10–21%, and that of TNF-α by about 20–26%, as shown in Figure 2.

**Figure 3:**
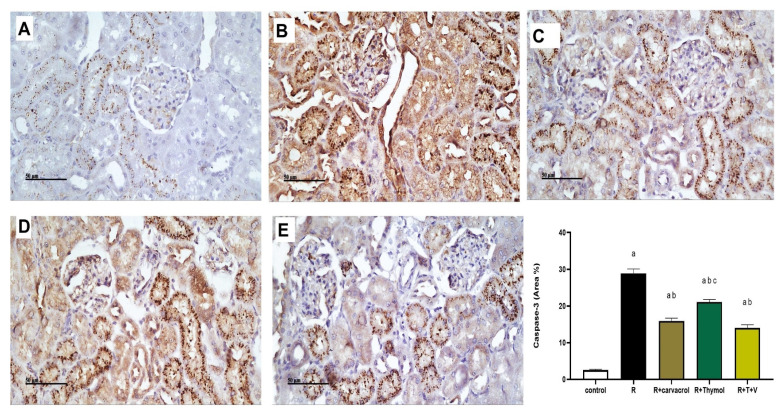
Microscopic Pictures of Renal Caspase-3 Expression in All Experimental Groups by Immunohistochemical Staining (**A**–**E)** and its Statistical Analysis as Area % of Immune-Positive staining. Renal sections showed a minimal Caspase-3 expression in the control group (**A**), an extensive expression in the irradiated group (**B**) and moderate expression in carvacrol- and/or thymol-treated groups (**C**–**E**). (**A**–**E**): x200, bar 200 μm. Data are presented as mean ± SEM. ^a^ *p* < 0.05 versus control group, ^b^ *p* < 0.05 versus irradiated group, ^c^ *p* < 0.05 versus R+T+V group using one-way ANOVA followed by Tukey–Kramer as post hoc test. R: γ-irradiation; V: Carvacrol; T: Thymol.

Figure 3 shows Caspase-3 immunoexpression in different groups and the results showed intense immunostaining in rat sections of the irradiated group. The % of immunostaining in the irradiated group was about 15 times greater than that in the control group. On the contrary, Caspase-3 expression was significantly reduced in either carvacrol- or thymol- or their combination-treated groups by approximately 60%, 80% and 50%, respectively, when compared to the irradiated group (Figure 3). However, neither carvacrol nor thymol administration -normalized the Caspase-3 immunoexpression levels compared to the control group.

### 3.4. Carvacrol and/or Thymol Suppresses the γ-Irradiation-Induced Upregulation of IGF-1 and CGRP Expressions

**Figure 4:**
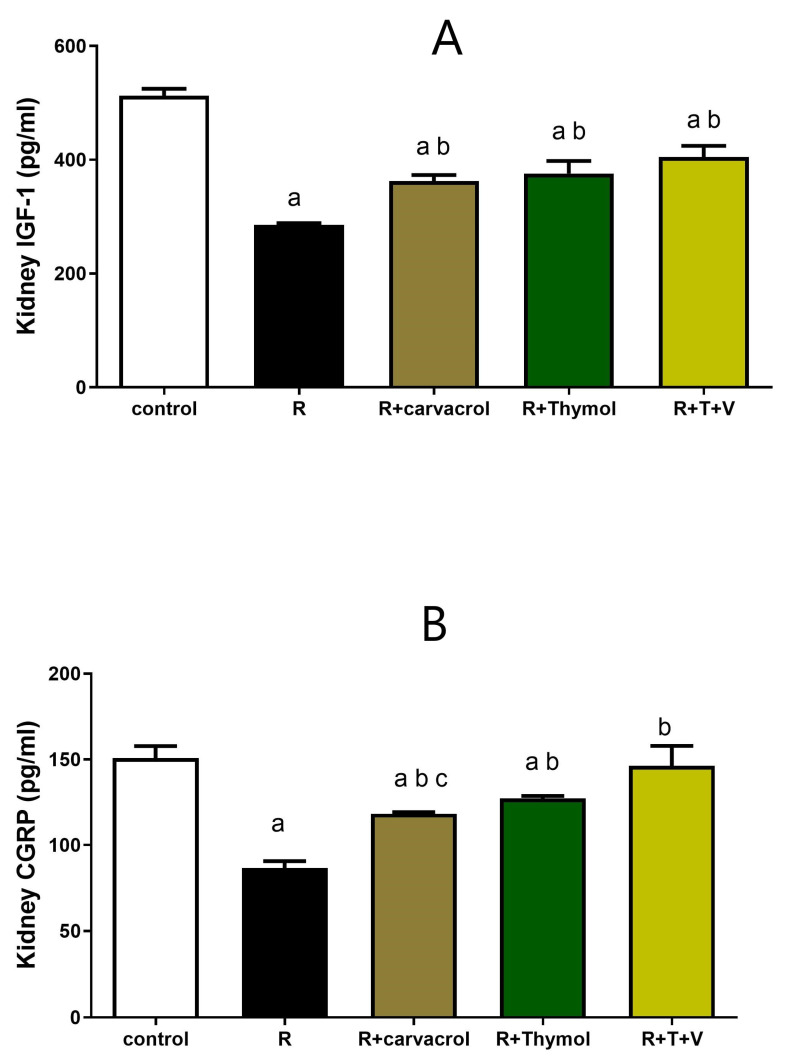
Impact of Carvacrol and/or Thymol on Renal Expressions of Insulin-like Growth Factor-1 (IGF-1) (**A**) and Calcitonin Gene-Related Peptide (CGRP) (**B**) in γ-irradiated Rats. Data are presented as mean ± SEM. ^a^ *p* < 0.05 versus control group, ^b^ *p* < 0.05 versus irradiated group, ^c^ *p* < 0.05 versus R+T+V group using one-way ANOVA followed by Tukey–Kramer as post hoc test. R: γ-irradiation; V: Carvacrol; T: Thymol.

As shown in Figure 4, γ-irradiation induced an intense decline in the renal expression of both IGF-1 and CGRP reaching approximately 50% and 57% that of the control group. However, pre-treatment of irradiated rats with carvacrol, thymol, or their combination significantly counteracted these changes and increased the renal expression of IGF-1 by approximately 126%, 132%, 140% and CGRP by approximately 135%, 144%, and 167%, respectively, compared to the irradiated group (Figure 4). However, carvacrol- or thymol-treated groups still showed a significant value of IGF-1 when compared to the control group (Figure 4A). With regard to the renal expression of CGRP, the administration of thymol or thymol and carvacrol combined to irradiated rats caused no significant change when compared to the control group (Figure 4B).

### 3.5. Docking Results of Carvacrol and Thymol

**Figure 5:**
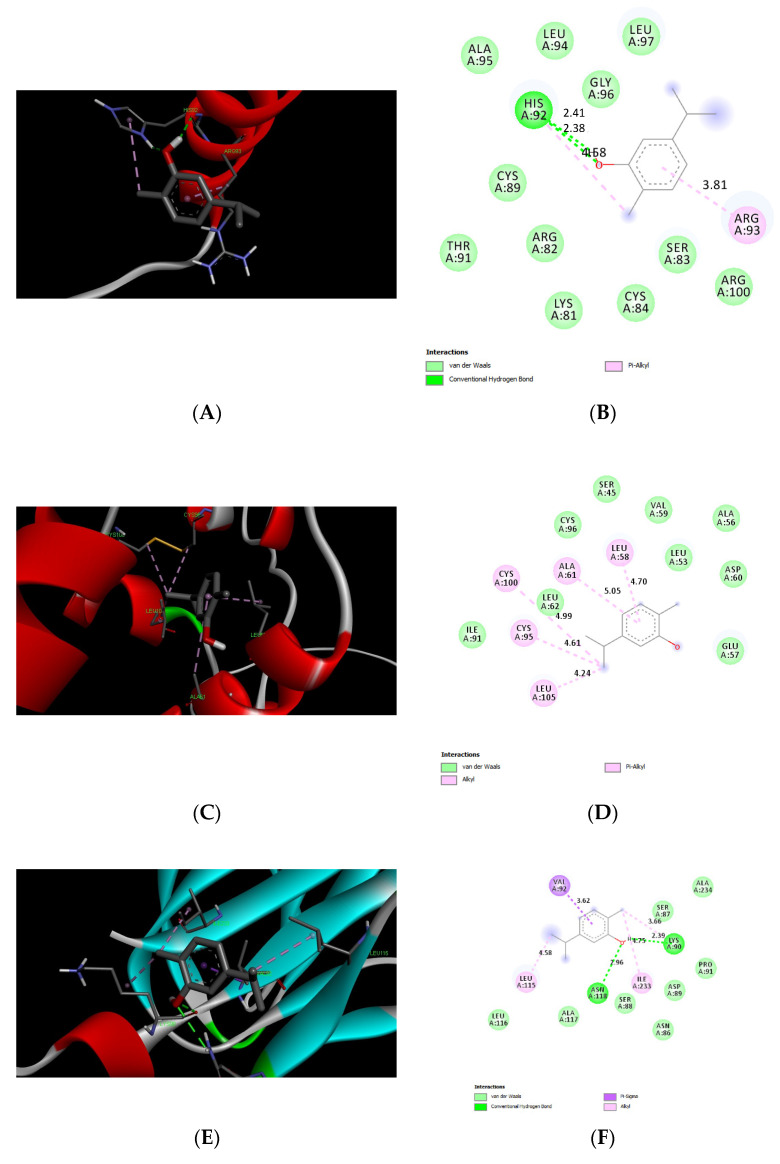

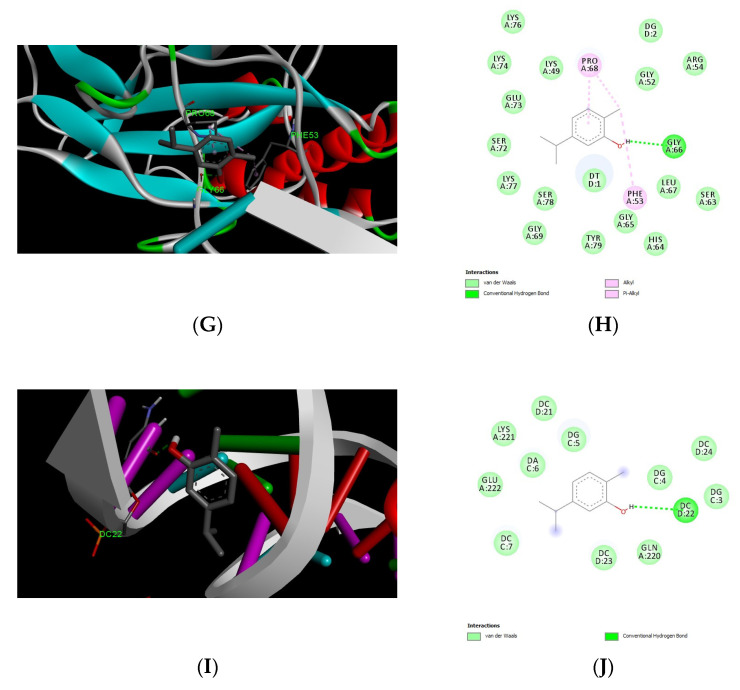
Three-dimensional (**left panels**) and two-dimensional (**Right Panel**) molecular docking poses showing interactions of carvacrol with binding sites of CGRP (**A**,**B**), IGF-1 (**C**,**D**), TNF-α (**E**,**F**), NF-kB1 (**G**,**H**), and p65 (**I**,**J**).

**Figure 6:**
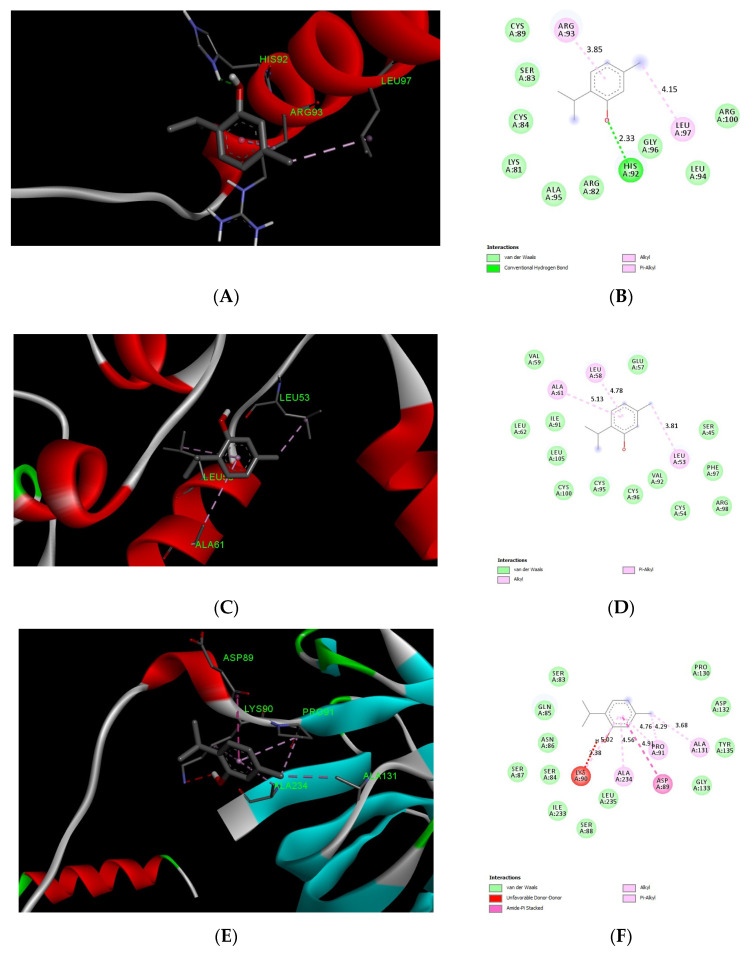

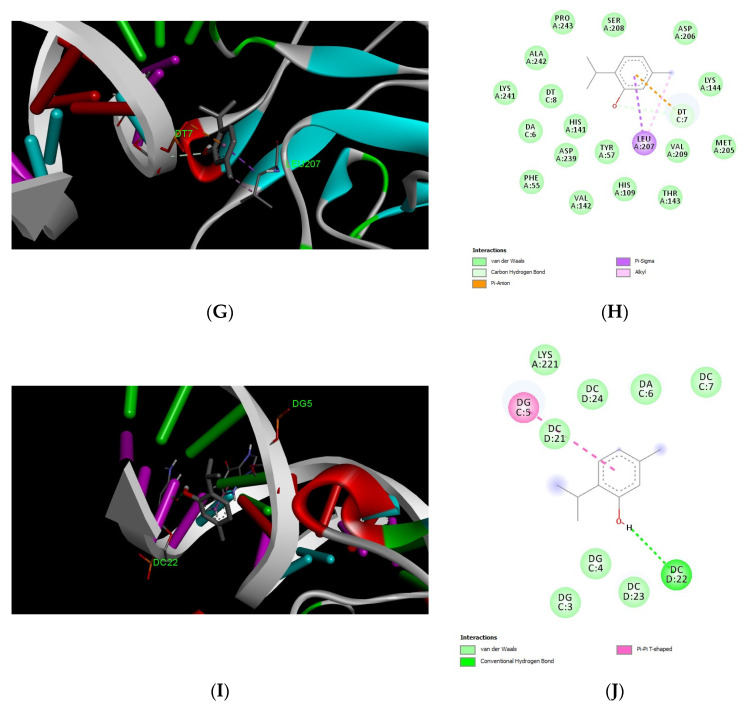
Three-dimensional (**left panels**) and two-dimensional (**Right Panel**) molecular docking poses showing interactions of thymol with binding sites of CGRP (**A**,**B**), IGF-1 (**C**,**D**), TNF-α (**E**,**F**), NF-kB1 (**G**,**H**), and p65 (**I**,**J**).

We tested molecular docking between carvacrol or thymol and CGRP, IGF1, TNF-α, NF-κB1, and p65 subunit. The protein IDs, binding affinities, types of interactions, and the amino acids involved in these interactions are shown in Table 2 and Table 3. The results showed comparable affinities of carvacrol or thymol docking with target proteins of less than −4.4 kcal/mol, which indicates promising stable binding (Figure 5 and Figure 6).

## 4. Discussion

Radiotherapy is considered one of the approved curative treatments for cancer. However, the unavoidable exposure of normal organs to radiation became an inevitable source of serious toxicity and suboptimal tumor control. Despite increasing research on the treatment of RN, the underlying molecular mechanism has not yet been fully elucidated. Therefore, understanding the mechanisms underlying the nephroprotection against acute RN is vital to improve survival in cancer patients receiving radiotherapy. To the best of our knowledge, the present study is the first to unveil the potential role of growth factor IGF-1 and CGRP in radioprotection afforded by carvacrol or thymol and their combination against γ-irradiation-mediated acute nephropathy in rats.

In the present study, a single dose of whole-body γ-irradiation (6 Gy) resulted in increased BUN levels and renal oxidative and inflammatory indices. γ-irradiation also induced severe tubular degeneration, glomerular atrophy, necrobiotic changes, and karyopyknosis in different tubular segments, which were consistent with increased renal indices, as previously reported [10,20,52]. These findings indicate that the accumulation of BUN occurs early as a result of the inability of the kidney to clear nitrogenous substances owing to severe morphological damage and functional disability in the kidney [53], which is consistent with previous studies [54]. Clinically, Cohen et al. suggested that azotaemia may be detectable before clinical symptoms of acute RN occur [55]. Additionally, in our study, the administration of carvacrol or thymol and their combination markedly alleviated nephrotoxic damage, supporting their promising nephroprotective role, as previously reported [37,38,56].

In addition to evaluating markers of nephrotoxicity, the mechanisms underlying carvacrol and thymol radioprotection have also been thoroughly investigated. Oxidative stress, inflammation, and apoptosis are among the confirmed molecular pathomechanisms to γ-irradiation-mediated acute kidney injury (AKI) [17,18,19]. Ionizing radiation causes the generation of ROS in renal tissues promptly after irradiation, which outweighs the antioxidant pathways, and injures cellular macromolecules, e.g., lipids, proteins or DNA [10,57,58]. In the present study, irradiated rats showed a significant oxidative stress in the renal tissues, as indicated by a significant increase in lipid peroxidation and depletion of GSH. These findings are consistent with those reported in previous studies, showing clear evidence that oxidative stress plays an important role in promptly damaging the DNA after irradiation [10,57]. In our study, both carvacrol, thymol, and their combination alleviated renal oxidative stress induced by γ-irradiation, which is consistent with the results of recent studies reporting the radioprotective effects of thymol and carvacrol through an antioxidant mechanism [29,32,35]. In this context, studies have documented the mitigation of radiation-induced oxidative stress when the antioxidative substances were administrated before γ-irradiation, supporting our findings [59,60].

Moreover, it is well-documented that ROS can activate both the inflammatory and apoptotic pathways [61,62,63,64,65], and inflammatory response is one of the immediate signaling events involved in radiation-induced AKI [66]. In our study, irradiation of rats with a single dose of whole-body γ-irradiation (6 Gy) resulted in the upregulation of renal expression levels of NF-κB, TNF-α, and Caspase-3, confirming the inflammatory and apoptotic responses induced by irradiation. Ionizing radiation induces oxidative stress leading to a DNA DSB. This acute DSB in the DNA triggers an immediate cell death in the kidney [10,16,18]. Even in those cells which did not die from the acute DNA damage, mis-repaired DSBs might induce cell death or cellular senescence and inflammation in the long term [67,68]. Although there is scarcity of data on active inflammation in RN, the inhibition of the renal expressions of NF-κB and TNF-α was found to be involved in the protective effect of montelukast against RN in a dose-dependent manner [60,69]. In our study, the administration of carvacrol or thymol and their combination substantially alleviated the renal inflammation and apoptosis, which is consistent with the results of recent studies [70,71]. Therefore, we suggest that carvacrol and thymol may mitigate the γ-irradiation-induced inflammatory or apoptotic response by rebalancing the oxidative stress status in renal tubular cells.

Indeed, a key bi-directional influence has been documented between TNF-α and CGRP in the nervous and immune systems. Moreover, the involvement of CGRP in the generation of both pro- and anti-inflammatory immune responses is known in some inflammatory models and immune conditions, e.g., diabetes [72], sepsis [73], Crohn’s Disease [74], and ulcerative colitis [75]. In rats, treatment with a CGRP antagonist worsened the dextran sulfate sodium (DSS)-induced colitis suggesting a protective role for CGRP in colitis [76]. A study by Khan and his co-workers suggested that TNF-α increased the CGRP release from sensitized capsaicin-sensitive neurons in rats [77]. On the other hand, treatment with CGRP antagonists inhibited the capsaicin-mediated reduction in TNF-α release that was induced by lipopolysaccharides [78], suggesting a regulatory role of CGRP on TNF-α; i.e., CGRP is able to downregulate TNF-α in vivo [79]. Additionally, the inhibition of the CGRP release worsened the gastric mucosal damage through upregulation of TNFα serum levels [80]. This study is the first to suggest that the CGRP- TNF-α loop plays a crucial role in the γ-irradiation-mediated inflammation; i.e., γ-irradiation-induced renal CGRP downregulation might have led to an increase in renal TNF-α, an effect which was reversed in the carvacrol- and thymol-treated groups, explaining their nephroprotective mechanisms.

The present study aimed to explore the involvement of CGRP signaling and IGF-1 in the protective mechanisms against γ-irradiation-induced acute RN. IGF-1 and CGRP have recently been reported to play a role in nephroprotective mechanisms of different modalities [25,81,82]. Indeed, CGRP was shown to improve renal hemodynamics through increasing the renal cyclic AMP levels in a dose-dependent manner both in vitro [22] and in vivo [23]. This improvement in renal blood flow was linked to its nephroprotective mechanism in a model of deoxycorticosterone-salt hypertension in mice [82]. Additionally, carvedilol might have improved the blood pressure in spontaneous hypertensive rats through upregulation of renal expression of CGRP [83]. The present study was the first to demonstrate that calcitonin gene-related peptide expression was significantly decreased in γ-irradiation-mediated acute RN; however, its level was nearly normalized in the carvacrol- and/or thymol-pre-treated groups. IGF-1 upregulation has been linked mutually to the CGRP signaling in some settings, and both are interrelated to the inflammatory mediators in cross-talk relationships [27,28]. Moreover, the depletion of growth factors and their receptors has recently been shown to play a role in RN. In a rat model, γ-irradiation resulted in a downregulation of the IGF-1/IGF-1 receptor pathway [84]. Also, Yasuda et al. [85] demonstrated that the exogenous administration of recombinant human IGF-1 attenuated the renal damage induced by γ-irradiation in rats.

Additionally, the in silico docking studies provide evidence for the potential binding affinities between carvacrol or thymol and the binding pockets of target proteins CGRP, IGF-1, TNF-α, and NF-κB. These results indicate the promising anti-inflammatory as well as reno-protective effects of carvacrol and thymol. Furthermore, our recent publication by Badr et al. supported the antioxidative and anti-inflammatory activities of CAR in an experimental model of a gastric ulcer with a good binding affinity to TNF-α [86].

Collectively, this study is the first to provide evidence for IGF-1 as well as CGRP signaling downregulation by γ-irradiation, an effect which was significantly attenuated by both carvacrol and thymol pre-treatment. However, the effects of carvacrol or thymol on the cytotoxic activity of γ-irradiation still need to be investigated and further clinical trials are highly warranted.

## 5. Conclusions

This study demonstrated that carvacrol and/or thymol pre-treatment provides a marked nephroprotection against γ-irradiation-induced acute nephropathy. This nephroprotective effect could be partially attributed to its antioxidative, anti-inflammatory, and antiapoptotic activities. Moreover, carvacrol and/or thymol counteracted the RN by increasing the expressions of growth factors IGF-1 and CGRP that had declined following a single dose of γ-irradiation This finding may pave the way for investigating the effects of carvacrol and/or thymol in other pathological conditions where IGF-1 and CGRP depletion is involved. Additionally, this study shed a light on the possible role of CGRP- TNF-α loop downregulation in acute RN which was reversed by both carvacrol and thymol treatment. Collectively, our findings open a new avenue for using carvacrol and thymol to improve the therapeutic outcome of γ-irradiation.

## Figures and Tables

**Table 1:** Impact of Carvacrol and/or Thymol on γ-irradiation (R)-Induced Changes in Blood Urea Nitrogen and Oxidative Stress.

Treated Groups	BUN (mg/dL)	Glutathione (mM/mg Protein)	TBARS (nmol/mg Protein)
Control	0.033 ± 0.004	0.109 ± 0.013	8.050 ± 0.645
Irradiated	0.095 ^a^ ± 0.020	0.003 ^a^ ± 0.005	12.330 ^a^ ± 0.551
R+V	0.048 ^a,b^ ± 0.004	0.195 ^a,b,c^ ± 0.019	8.020 ^b^ ± 0.560
R+T	0.042 ^a,b^ ± 0.004	0.209 ^a,b,c^ ± 0.024	7.484 ^b^ ± 0.317
R+T+V	0.067 ^a,b^ ± 0.005	0.304 ^a,b^ ± 0.016	6.660 ^b^ ± 0.528

Data are presented as mean ± SEM (n  =  5). ^a^ *p* < 0.05 versus control group, ^b^ *p* < 0.05 versus irradiated group, ^c^ *p* < 0.05 versus R+T+V group using one-way ANOVA followed by Tukey–Kramer as post hoc test. R: **γ**-irradiation; BUN: Blood Urea Nitrogen; T: Thymol; TBARS: Thiobarbituric acid reactive substances, V: Carvacrol.

**Table 2:** Binding affinities and types of interaction of carvacrol with target protein.

Protein Name	ID	∆G (Binding Affinity Kcal/mol)	Amino Acids in Interaction	Type of Interaction
CGRP	AF_P01256_F1	−4.4	His 92	Pi-alkyl H-bond
			ARG 93	Pi-alkyl
IGF1	AF-P08025-F1	−4.5	Leu 58	Pi-alkyl
			Ala 61	Pi-alkyl
			Cys 100	alkyl
			Cys 95	Alkyl
			Leu 105	alkyl
TNF-α	AF-P16599-F1	−4.8	Val 92	Pi-sigma
			Leu 115	alkyl
			Asn 118	H-Bond
			Lys 90	H-Bond
			Ile 233	Alkyl
NF-κB	1nfk	−6.2	Pro 68	alkyl
				Pi-alkyl
			Phe 53	Pi-alkyl
			Gly 66	h-bond
P65	1vkx	−5.5	C 22	h-bond

**Table 3:** Binding affinities and types of interaction of thymol with target protein.

Protein Name	ID	∆G (Binding Affinity Kcal/mol)	Amino Acids in Interaction	Type of Interaction
CGRP	AF_P01256_F1	−4.4	Leu 97	Alkyl
			Arg 93	Pi-alkyl
			His 92	h-bond
IGF1	AF-P08025-F1	−4.5	LUE 53	Alkyl
			Leu 58	Pi-alkyl
			Ala 61	
TNF-α	AF-P16599-F1	−4.8	Lys 90	Pi-alkyl
			Pro 91	Alkyl
				Pi-alkyl
			Ala 234	Pi-alkyl
NF-κB	1nfk	−5.9	T 7	Pi-anion
				Carbon hydrogen
			Leu 207	Pi-sigma
				alkyl
P65	1vkx	−5.4	G 5	Pi-pi t shaped
			C 22	h-bond

## Data Availability

The authors confirm that the data supporting the findings of this study are available within the article.

## Abbreviations

AKI: acute kidney injury; BUN: blood urea nitrogen; CGRP: Calcitonin Gene-related Peptide; NF-kB: nuclear factor-kB; ROS: reactive oxygen species; TBARS: thiobarbituric acid reactive substances.
